# Overview and assessment of the histochemical methods and reagents for the detection of β-galactosidase activity in transgenic animals

**DOI:** 10.1007/s12565-015-0300-3

**Published:** 2015-09-22

**Authors:** Stefan Trifonov, Yuji Yamashita, Masahiko Kase, Masato Maruyama, Tetsuo Sugimoto

**Affiliations:** Department of Anatomy and Brain Science, Kansai Medical University, 2-5-1 Shin-machi, Hirakata, Osaka 573-1010 Japan

**Keywords:** β-galactosidase, *lacZ*, Nitroblue tetrazolium, Salmon-gal, X-gal

## Abstract

Bacterial β-galactosidase is one of the most widely used reporter genes in experiments involving transgenic and knockout animals. In this review we discuss the current histochemical methods and available reagents to detect β-galactosidase activity. Different substrates are available, but the most commonly used is X-gal in combination with potassium ferri- and ferro-cyanide. The reaction produces a characteristic blue precipitate in the cells expressing β-galactosidase, and despite its efficiency in staining whole embryos, its detection on thin tissue sections is difficult. Salmon-gal is another substrate, which in combination with ferric and ferrous ions gives a reddish-pink precipitate. Its sensitivity for staining tissue sections is similar to that of X-gal. Combining X-gal or Salmon-gal with tetrazolium salts provides a faster and more sensitive reaction than traditional β-galactosidase histochemistry. Here, we compare the traditional β-galactosidase assay and the combination of X-gal or Salmon-gal with three tetrazolium salts: nitroblue tetrazolium, tetranitroblue tetrazolium and iodonitrotetrazolium. Based on an assessment of the sensitivity and specificity of the different combinations of substrates, we are proposing an optimized and enhanced method for β-galactosidase detection in histological sections of the transgenic mouse brain. Optimal staining was obtained with X-gal in combination with nitroblue tetrazolium, which provides a faster and more specific staining than the traditional X-gal combination with potassium ferri- and ferro-cyanide. We recommend the X-gal/nitroblue tetrazolium staining mixture as the first choice for the detection of β-galactosidase activity on histological sections. When faster results are needed, Salmon-gal/nitroblue tetrazolium should be considered as an alternative, while maintaining acceptable levels of noise.

## *lacZ* as a reporter gene in molecular biology

Reporter genes are used in myriad transgenic experiments, from *Drosophila* to mammals, during embryonic development and in the postnatal period. One of the most commonly used reporter genes in molecular biology is the β-galactosidase gene (*lacZ*) of *Escherichia coli* (Beckwith 1980; Cui et al. 1994; Takahashi et al. 2000; Burn 2012). In the mouse, *lacZ* has been used to identify the *cis*-acting DNA elements important for the regulation of gene expression, by knocking it into the endogenous gene or locus of interest or by randomly integrating into the genome a plasmid containing *lacZ* under the control of the regulatory elements of interest (Goring et al. 1987; Bonnerot and Nicolas 1993; Sekerková et al. 1997). The *lacZ* gene is frequently used in the detection of genetic recombination events by Cre-loxP and the detection of embryonic stem cell derivatives in cell fate mapping experiments (Petit et al. 2005; Joyner and Zervas 2006; Watson et al. 2008). The use of Cre recombinase allows tissue-specific and/or temporal control of *lacZ* expression. Cre-expressing mice are often used in combination with the Rosa26R mouse line, which has a ubiquitously expressed Cre-inducible *lacZ* (Soriano 1999).

The complete sequencing of human and mouse genomes led to the next important goal, functional analysis of more than 20,000 protein-coding genes. To help accomplish this tremendous task, the trans-NIH Knockout Mouse Project (KOMP) was developed. The objective of the KOMP is to produce some 21,000 embryonic stem cell lines that have one of the 21,000 protein-coding mouse genes knocked out. The major targeting pipeline of this international mouse knockout program is to generate *lacZ*-tagged null mutations in every protein-coding gene in the mouse (Skarnes et al. 1992; Skarnes et al. 2011). Thousands of genes have been successfully targeted in C57BL/6N embryonic stem cells using this technology, and many knockout mice expressing β-galactosidase of *Escherichia coli* as a reporter are readily available.

## Standard β-galactosidase histochemical reaction

**Fig. 1:**
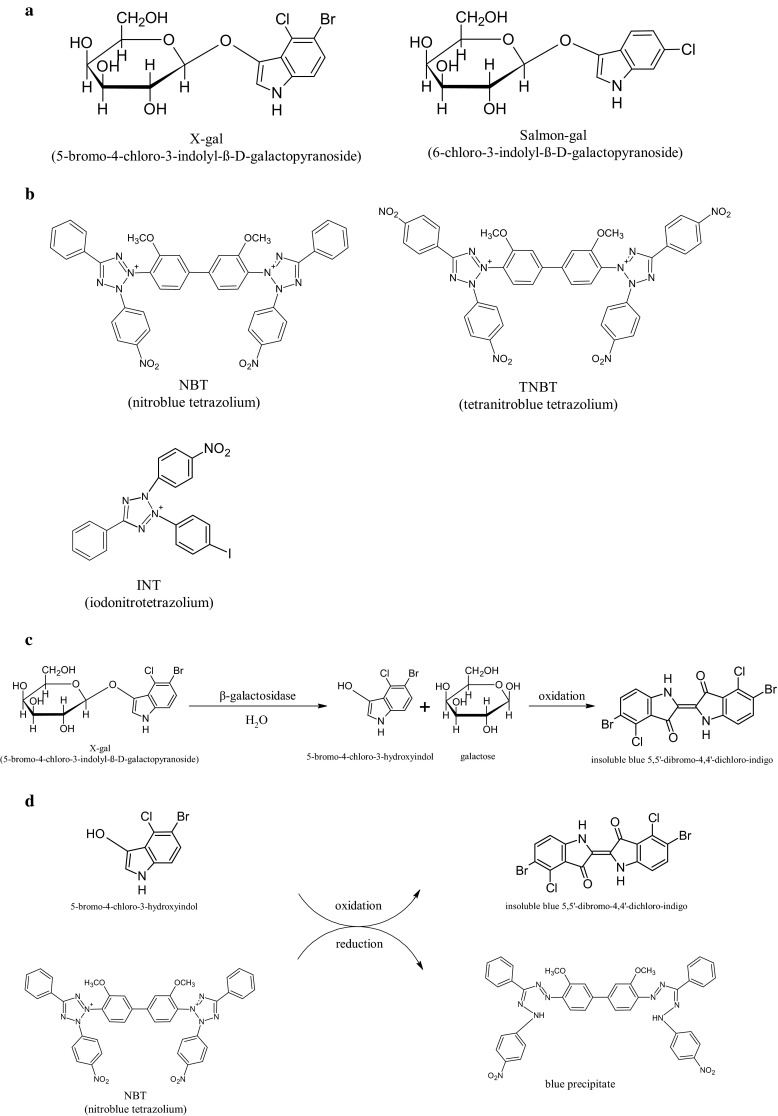
Diagram showing **a** the chemical formulas of the two most popular β-galactosidase substrates: X-gal (5-bromo-4-chloro-3-indolyl-β-D-galactopyranoside) and Salmon-gal (S-gal, 6-chloro-3-indolyl-β-D-galactopyranoside); **b** the structure of NBT (nitroblue tetrazolium), TNBT (tetranitroblue tetrazolium) and INT (iodonitrotetrazolium); **c** the mechanism of the traditional β-galactosidase staining utilizing X-gal in combination with ferric and ferrous ions; **d** the mechanism of the modified β-galactosidase staining with NBT as substitute for ferric and ferrous ions

β-galactosidase is a tetramer of four identical polypeptide chains, each comprising 1023 amino acids (Juers et al. 2012). It cleaves the disaccharide lactose to form glucose and galactose. It can also catalyze the transgalactosylation of lactose to allolactose, which binds to the *lacZ* repressor and regulates the amount of β-galactosidase in the cell. β-galactosidase is best recognized for its reaction with X-gal (5-bromo-4-chloro-3-indolyl-β-D-galactopyranoside), a soluble colorless glycoside consisting of galactose linked to a substituted indole (Fig. 1a). β-galactosidase has high specificity for the galactose part and hydrolyzes X-gal. In this first step, a soluble colorless indolyl group is released from X-gal (Fig. 1c). In the second step of the reaction, two indolyl moieties dimerize and are oxidized to give an insoluble, intensely blue indigo precipitate (Fig. 1d) (Cotson and Holt 1958; Pearson et al. 1963). Ferric and ferrous ions, which serve as electron acceptors, greatly facilitate the dimerization and oxidation of the indolyl groups (Lojda 1970). There are various chromogenic substrates for X-gal. Some of them yield soluble colored or fluorescent products and can be used to quantify β-galactosidase activity in cells. However, chromogenic substrates that yield a precipitated product like X-gal are needed for localization studies of β-galactosidase expression in histological sections or whole embryos and organs (Pearson et al. 1963).

Fluorescent substrates that are lipophilic analogs of fluorescein di-β-D-galactopyranoside were developed to facilitate the detection of *lacZ* gene expression in living cells (Zhang et al. 1991). The combination of fluorescent substrates with classical immunofluorescence for co-localization analysis may be a valuable tool in gene expression and cell tracing experiments. The use of a titanium-doped sapphire laser for photoactivation of the X-gal precipitate has been reported (Matei et al. 2006), which transforms the light-absorbing X-gal precipitate into an intensely fluorescent product suitable for confocal microscopy. A recent study (Levitsky et al. 2013) revealed a much simpler technique for obtaining direct confocal images of X-gal fluorescence, wherein the authors reported that after excitation of the X-gal precipitate with a helium–neon laser at 633 nm, they were able to detect an emission signal in the 650-770 nm range. This finding, combined with the introduced mathematically based optical correction, allows high-quality confocal images to be obtained from X-gal stained sections without photoactivation.

There are several chromogenic substrates that produce colored precipitates and can replace X-gal, including Salmon-gal (S-gal, 6-chloro-3-indolyl-β-D-galactopyranoside) (Fig. 1a), Magenta-gal (5-bromo-6-chloro-3-indolyl-β-D-galactopyranoside) and Bluo-gal (5-bromo-3-indolyl-β-D-galactopyranoside) (Aguzzi and Theuring 1994; Schmidt et al. 1998; Kishigami et al. 2006; Sundararajan et al. 2012). Salmon-gal in combination with ferric and ferrous ions has been reported to be more sensitive in early embryos (Kishigami et al. 2006).

## Tetrazolium salts are an enhancer of the β-galactosidase histochemical reaction

A faster and more sensitive alternative for the detection of β-galactosidase activity in mouse embryos using a combination of Salmon-gal and tetranitroblue tetrazolium was recently reported (Sundararajan et al. 2012). Tetrazolium salts, like nitroblue tetrazolium (NBT), tetranitroblue tetrazolium (TNBT) and iodonitrotetrazolium (INT), can be substituted for potassium ferri- and ferro-cyanide as final electron acceptors, and precipitate when reduced to form colored formazan compounds (Fig. 1b, d) (Altman 1976). Phenazine methosulfate can further increase the reaction rate by quantitatively reducing tetrazolium salts. X-gal combined with NBT produces a deep dark blue precipitate; in combination with INT it yields a dark orange-red precipitate, and together with TNBT, an intense dark brown precipitate (Altman 1976). The X-gal/TNBT combination was found to be more sensitive in tissue sections than the classic reaction of X-gal in combination with ferric and ferrous ions (Gugliotta et al. 1992). Salmon-gal mixed with NBT produces a dark purple precipitate; in combination with INT, it forms a dark brick-red precipitate, and with TNBT, a dark brown precipitate (Altman 1976; Sundararajan et al. 2012). The Salmon-gal/TNBT combination was found to be most sensitive for the detection of β-galactosidase in mouse embryos (Sundararajan et al. 2012).

## Comparison between the different histochemical methods for β-galactosidase detection

**Fig. 2:**
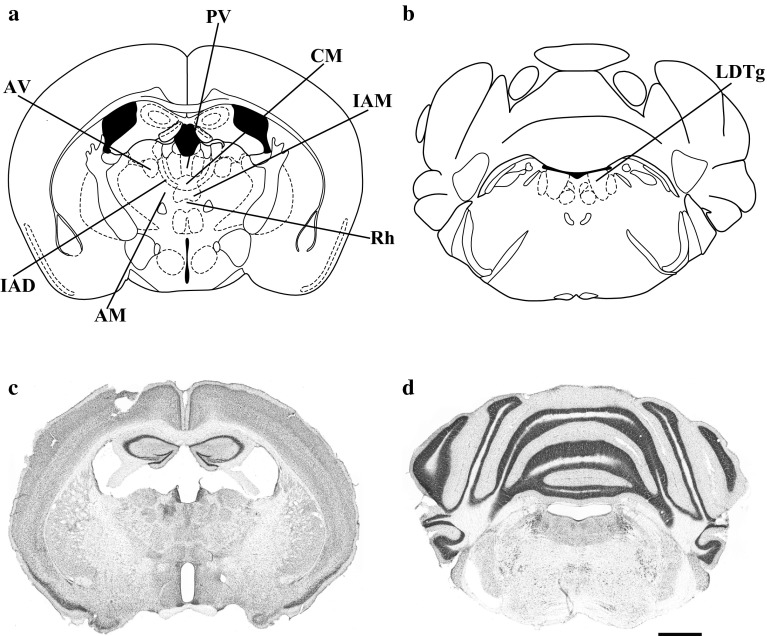
Line drawing presenting **a** the thalamic nuclei and **b** laterodorsal tegmental nuclei, which reveal the highest level of β-galactosidase expression in B6.Cg-Tg(Nes-cre)1Nogu mice. **c**, **d** Cresyl violet-stained coronal section of B6.Cg-Tg(Nes-cre)1Nogu mouse brain at the same level as in (**a**, **b**). *AM* anteromedial thalamic nucleus, *AV* anteroventral thalamic nucleus, *CM* central medial thalamic nucleus, *IAD* interanterodorsal thalamic nucleus, *IAM* interanteromedial thalamic nucleus, *PV* paraventricular thalamic nucleus, *Rh* rhomboid thalamic nucleus, *LDTg* laterodorsal tegmental nucleus. *Scale bar* 1 mm

The purpose of this review is to provide an overview of the current histochemical methods for detecting β-galactosidase (*lacZ* gene of *Escherichia coli*) expression in genetically engineered animals. Based on a comparison of their sensitivity and specificity, we are proposing an optimized and enhanced method for β-galactosidase detection in histological sections of the transgenic mouse brain. The experiments were carried out with 10-week-old male heterozygous B6.Cg-Tg(Nes-cre)1Nogu mice (No. RBRC02412), provided by RIKEN BioResource Center (BRC) through the National BioResource Project of the Ministry of Education, Culture, Sports, Science, and Technology (MEXT), Japan. These mice were chosen over the ubiquitously β-galactosidase-expressing Rosa26 mice because the level of expression of the *lacZ* gene is lower, which makes them suitable for a comparison of the sensitivity of different substrates. These transgenic mice express Cre recombinase under the control of the Nestin promoter/enhancer. The Nestin-Cre transgene contains rat nestin promoter, Cre recombinase gene and IRES (internal ribosomal entry site)-*lacZ*-polyA (Mishina and Sakimura 2007; Tanaka et al. 2012). C57BL/6J wild-type male mice of the same age were used as controls. All experiments were performed in compliance with the National Institutes of Health Guide for the Care and Use of Laboratory Animals (NIH Publication No. 80-23, revised 1996) and the Kansai Medical University local guidelines for animal experimentation (issued 9 March 1999; registration number of the current research proposal, 12-064; permit number, 25-055). The study had the full approval of the Institutional Committee for Animal Experimentation. All efforts were made to reduce the number of animals and their suffering. The animals were deeply anesthetized with sodium pentobarbital (100 mg/kg, i.p.) and transcardially perfused with 0.9 % saline solution (1 ml/g b.wt.), followed by freshly prepared 4 % paraformaldehyde, 1.25 mM EGTA and 2 mM MgCl_2_ in 0.1 M phosphate buffer (PB, pH 7.5; 2 ml/g b.wt.). The brains were dissected, postfixed by immersion in the same fixative for 2 h at 4 °C, and then transferred to 30 % sucrose in 0.1 M PB (pH 7.5) at 4 °C until they sank. The brains were then frozen on a sliding microtome, and 40-µm-thick sections were cut on the coronal plane. The tissue sections were collected in cryoprotection buffer (30 % sucrose, 30 % ethylene glycol, 50 mM PB) and stored at −20 °C until processing. They were then washed three times in rinse solution containing 0.02 % Nonidet^®^ P-40, 0.01 % sodium deoxycholate, 2 mM MgCl_2_, 1.25 mM EGTA and 0.1 M phosphate buffer (pH 7.5) for 15 min each. The staining solution consisted of either 1 mg/ml X-gal (Nacalai Tesque, Inc., Kyoto, Japan) or 1 mg/ml Salmon-β-D-gal (Biosynth International, Inc., Itasca, IL, USA), in combination with (1) 5 mM K_3_Fe(CN)_6_ and 5 mM K_4_Fe(CN)_6_, (2) 0.4 mg/ml NBT (Roche Diagnostics, Mannheim, Germany), (3) 0.2 mg/ml TNBT (Sigma-Aldrich, St. Louis, MO, USA), or (4) 0.8 mg/ml INT (Tokyo Chemical Industry Co., Ltd, Tokyo, Japan) respectively, in rinse solution at 37 °C, protected from light. Tetrazolium salts were dissolved in 70 % dimethylformamide. The staining reaction was monitored, and the optimal duration was determined for every combination of substrates. Finally, tissue sections were washed in 0.1 M phosphate-buffered saline (PBS), mounted on gelatin-coated slides, dehydrated with successive changes of ethanol (50, 75, 95, 100 and 100 % ethanol), clarified in three changes of xylene, and sealed with Canada balsam and a coverslip. The sections stained with a combination of X-gal or Salmon-gal and INT were washed in 0.1 M PBS, mounted on gelatin-coated slides and sealed with CC/Mount (Diagnostic Biosystems, Pleasanton, CA, USA) and a coverslip, thus avoiding dehydration and clearing in xylene, as INT precipitate dissolves completely in these organic solvents. Adjacent coronal sections were stained with cresyl violet to facilitate identification of nuclei and fiber tracts. The stained sections were viewed under a Nikon Eclipse E800 light microscope (Nikon Corp., Tokyo, Japan) under bright-field illumination. The nomenclature of nuclei and related fiber tracts was adopted from Paxinos and Franklin (2001) (Fig. 2a, b).

**Fig. 3:**
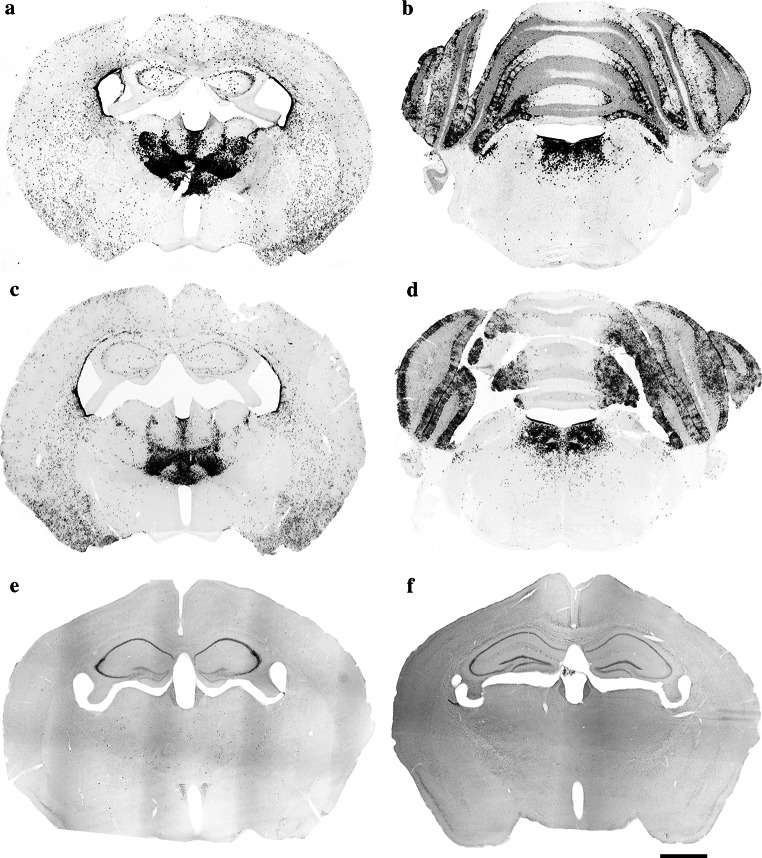
Low-magnification images showing the expression of β-galactosidase in the thalamic nuclei (**a**, **c**) and laterodorsal tegmental nuclei (**b**, **d**) of the B6.Cg-Tg(Nes-cre)1Nogu mouse brain, revealed by a combination of X-gal (**a**, **b**) or Salmon-gal (**c**, **d**) and nitroblue tetrazolium. Reaction time is 24 h for X-gal/nitroblue tetrazolium and 5 h for Salmon-gal/nitroblue tetrazolium. Control sections from wild-type mice at the level of the thalamus stained with X-gal (**e**) or Salmon-gal (**f**) and nitroblue tetrazolium. *Scale bar* 1 mm

**Fig. 4:**
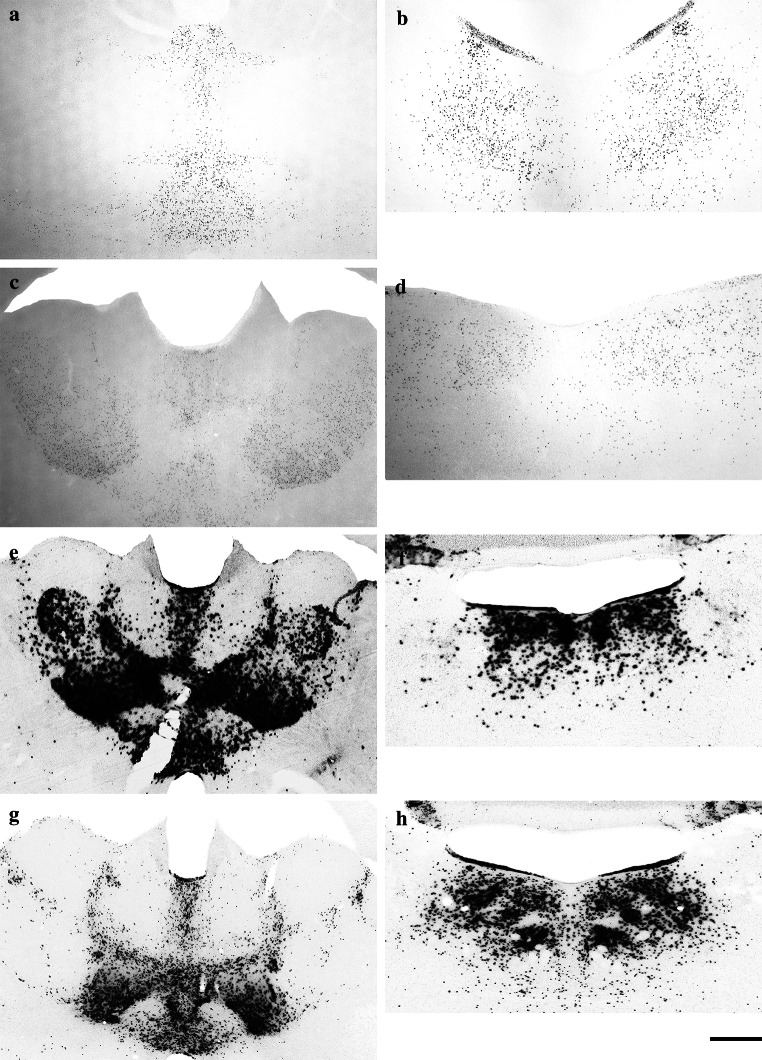
High-magnification images showing the expression of β-galactosidase in the thalamic nuclei (**a**, **c**, **e**, **g**) and laterodorsal tegmental nuclei (**b**, **d**, **f**, **h**) of B6.Cg-Tg(Nes-cre)1Nogu mouse brain, revealed by a combination of X-gal (**a**, **b**, **e**, **f**) or Salmon-gal (**c**, **d**, **g**, **h**) with potassium ferri- and ferro-cyanide (**a**, **b**, **c**, **d**) or nitroblue tetrazolium (**e**, **f**, **g**, **h**). Reaction time is 24 h for the X-gal/nitroblue tetrazolium, X-gal or Salmon-gal with potassium ferri- and ferro-cyanide staining mixtures, and 5 h for Salmon-gal/nitroblue tetrazolium. *Scale bar* 300 µm (**a**, **c**, **e**, **f**) and 200 µm (**b**, **d**, **g**, **h**)

According to the strain specifications, tissue-specific expression of Cre recombinase and β-galactosidase could be observed in the whole brain, spinal cord, testis and ovaries of B6.Cg-Tg(Nes-cre)1Nogu mice, but the age of the tested mice was not specified. Applying the most sensitive and specific histochemical procedure, we detected a specific pattern of β-galactosidase expression in the brain of adult male B6.Cg-Tg(Nes-cre)1Nogu mice (Figs. 3a, b, e, 4e, f). Intense labeling was seen in the granule cell layer of the accessory olfactory bulb; cells in the wall of the olfactory ventricle (data not shown) and lateral ventricle; paraventricular, anteroventral, interanterodorsal, interanteromedial, central medial, anteromedial, parafascicular and rhomboid thalamic nuclei (Figs. 3a, 4e); supramammillary nucleus; reticulotegmental nucleus of the pons (data not shown); Purkinje cells in the cerebellum; and laterodorsal tegmental nuclei (Figs. 3b, 4f). Moderate staining was observed in many neurons of the cerebral cortex, glomerular layer of the olfactory bulb, medial and lateral septal nuclei, and caudate putamen (data not shown). In the hippocampus, many moderately stained neurons could be seen dispersed in all of its layers. In CA1-3, some intensely stained pyramidal neurons were revealed, but the same neurons appeared positive also in the sections from the control wild-type animals. Some faint staining was seen in isolated cells in the paraventricular hypothalamic nucleus, and dispersed in many areas of wild-type mouse brain, largely coinciding with blood vessels. The choroid plexuses were also intensely stained in brain sections from wild-type mice. A similar pattern of staining, likely due to the detection of endogenous β-galactosidase activity in the brain of wild-type rats, was reported previously (Shimohama et al. 1989; Renfranz et al. 1991; Hatton and Lin 1992; Rosenberg et al. 1992; Sanchez-Ramos et al. 2000). The strongest labeling intensity was observed in the thalamus and laterodorsal tegmental nuclei of the brainstem in B6.Cg-Tg(Nes-cre)1Nogu mice, and staining in these regions was used to compare histochemical methods of β-galactosidase detection. Previous studies on wild-type rats have reported positive cells only in the anterodorsal thalamic nucleus and the medial habenula, which appear negative in B6.Cg-Tg(Nes-cre)1Nogu mice. Cresyl violet-stained sections corresponding to the same level were used for the identification of the nuclei of interest (Fig. 2b, c).

The expression of the *lacZ* gene in transgenic animals is most often detected using X-gal in combination with potassium ferri- and ferro-cyanide. This is a well-established staining assay that provides a clear blue precipitate that is easily detected. With staining of whole mouse embryos, the indigo precipitate is well marked, but on thin histological sections its pale blue color has low contrast and is difficult to detect and photograph, especially if *lacZ* expression is low (such as in heterozygote transgenic animals) (Fig. 4a, b). When it was used to stain sections from the brains of B6.Cg-Tg(Nes-cre)1Nogu mice, pale blue precipitate was visible only in the cells of the thalamic and laterodorsal tegmental nuclei. Moreover, when dehydrated in ethanol, cleared in xylene and mounted with xylene-based mounting medium (Canada balsam), the indigo product tended to diffuse in the surrounding tissue and to fade, as has been previously reported (Gugliotta et al. 1992). Thus it is not the most sensitive or fastest assay available for the detection of β-galactosidase (Table 1), and the blue color of the precipitate makes it less compatible with double-staining in situ hybridization or immunohistochemical experiments (Gugliotta et al. 1992; Kishigami et al. 2006; Sundararajan et al. 2012). Higher sensitivity and specificity have been reported for Salmon-gal in experiments involving embryos (Kishigami et al. 2006), with shorter incubation time, particularly in the early embryonic stages, leading the authors to conclude that Salmon-gal is capable of detecting lower levels of β-galactosidase activity, and its reddish-pink color makes it suitable for double-staining experiments in combination with in situ hybridization. Despite its higher efficacy in embryonic tissues, when it was used as a substrate in combination with ferric and ferrous ions to stain adult brain samples, Salmon-gal yielded staining patterns similar to those of X-gal under the same conditions and with incubation of the sections in the staining solution for up to 24 h. Visible precipitate was found in the cells of some thalamic nuclei and the laterodorsal tegmental nuclei (Fig. 4c, d). Staining of the control sections from the wild-type mouse brain with X-gal or Salmon-gal in combination with ferric and ferrous ions resulted in the absence of visible precipitate. Bluo-gal, another substrate for β-galactosidase, has also been reported as a highly sensitive alternative to X-gal (Aguzzi and Theuring 1994). It precipitates in the form of fine birefringent crystals, which when examined under polarized light, emit a strong signal consisting of yellow reflected light. This method was reported to have greatly enhanced sensitivity and optimal morphological resolution, but it requires the use of a polarized light microscope. Despite the widespread use of Salmon-gal and, in particular, X-gal for the detection of β-galactosidase activity, their low sensitivity in combination with ferric and ferrous ions can yield false-negative results and lead to erroneous conclusions when used to stain thin sections from rodent brains with lower expression of *lacZ* gene.

**Table 1:** Sensitivity and specificity of β-galactosidase X-gal and Salmon-gal substrates in combination with ferri- and ferro-cyanide or different tetrazolium salts

	X-gal				Salmon-gal			
	NBT	TNBT	INT	Fe	NBT	TNBT	INT	Fe
Sensitivity	***	***	*	*	***	***	*	*
Specificity	***	*	*	***	**	*	*	***

Sensitivity and specificity of the reaction are graded in three ranks, shown by asterisks: *** high, ** medium and * low

**Fig. 5:**
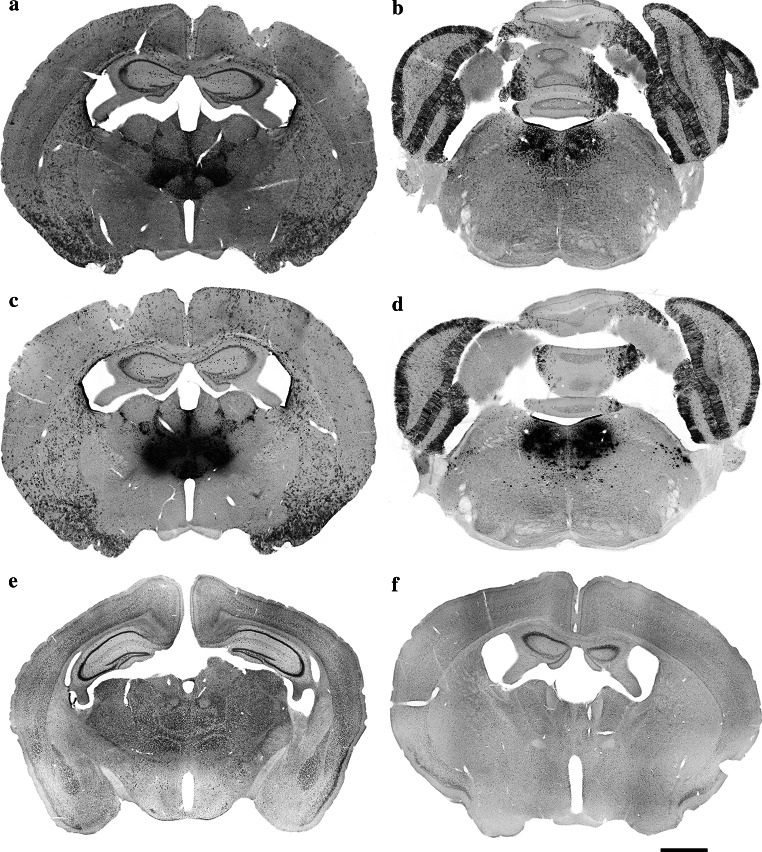
Low-magnification images showing the expression of β-galactosidase in the thalamic nuclei (**a**, **c**) and laterodorsal tegmental nuclei (**b**, **d**) of the B6.Cg-Tg(Nes-cre)1Nogu mouse brain, revealed by a combination of X-gal (**a**, **b**) or Salmon-gal (**c**, **d**) and tetranitroblue tetrazolium. Reaction time is 24 h for the X-gal/tetranitroblue tetrazolium and 5 h for Salmon-gal/tetranitroblue tetrazolium. Control sections from wild-type mice at the level of the thalamus stained with X-gal (**e**) or Salmon-gal (**f**) and tetranitroblue tetrazolium. *Scale bar* 1 mm

**Fig. 6:**
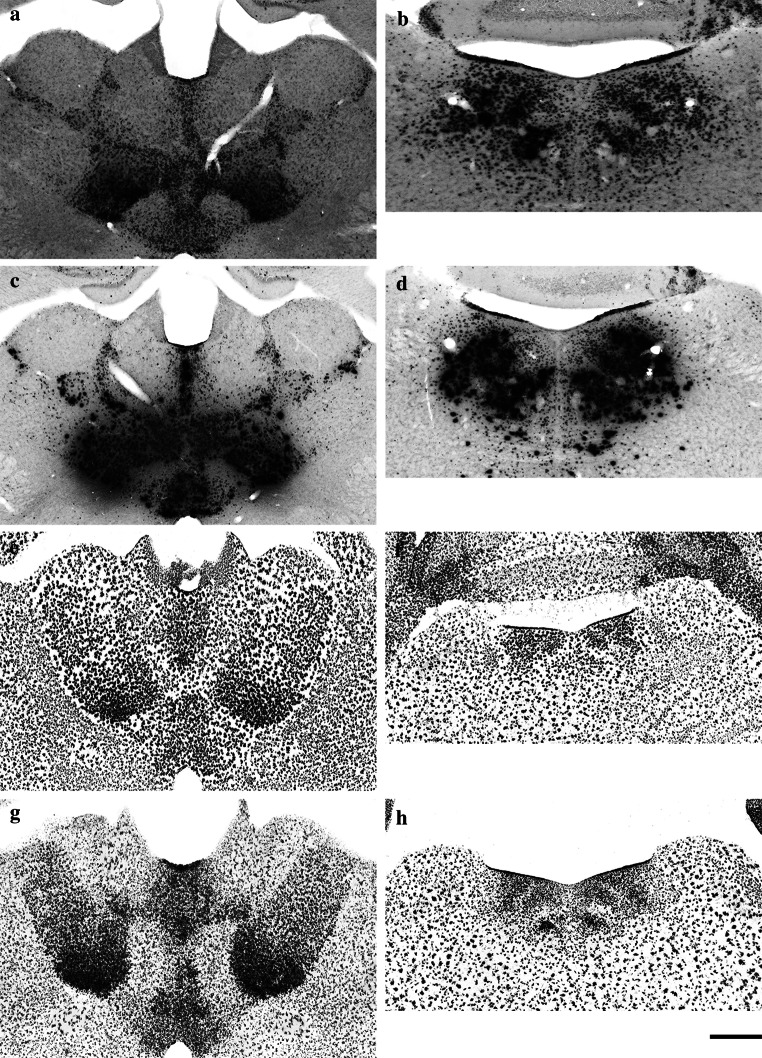
High-magnification images showing the expression of β-galactosidase in the thalamic nuclei (**a**, **c**, **e**, **g**) and laterodorsal tegmental nuclei (**b**, **d**, **f**, **h**) of the B6.Cg-Tg(Nes-cre)1Nogu mouse brain, revealed by a combination of X-gal (**a**, **b**, **e**, **f**) or Salmon-gal (**c**, **d**, **g**, **h**) and tetranitroblue tetrazolium (**a**, **b**, **c**, **d**) or iodonitrotetrazolium (**e**, **f**, **g**, **h**). Reaction time is 24 h for the X-gal/tetranitroblue tetrazolium, 5 h for Salmon-gal/tetranitroblue tetrazolium, and 48 h for the X-gal and Salmon-gal combinations with iodonitrotetrazolium. *Scale bar* 300 µm (**a**, **c**, **e**, **f**) and 200 µm (**b**, **d**, **g**, **h**)

**Fig. 7:**
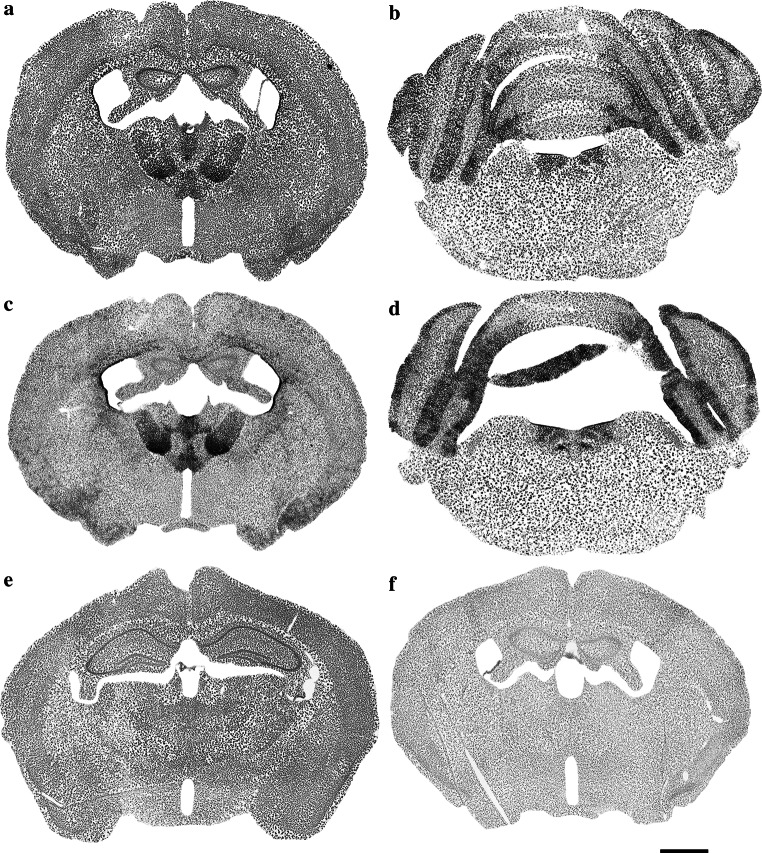
Low-magnification images showing the expression of β-galactosidase in the thalamic nuclei (**a**, **c**) and laterodorsal tegmental nuclei (**b**, **d**) of the B6.Cg-Tg(Nes-cre)1Nogu mouse brain, revealed by a combination of X-gal (**a**, **b**) or Salmon-gal (**c**, **d**) and iodonitrotetrazolium. Reaction time is 48 h. Control sections from wild-type mice at the level of the thalamus stained with X-gal (**e**) or Salmon-gal (**f**) and iodonitrotetrazolium. *Scale bar* 1 mm

In experimental embryology in mouse models, X-gal and Salmon-gal in combination with tetrazolium salts showed greater sensitivity for the detection of β-galactosidase activity, thus helping avoid false-negative results (Gugliotta et al. 1992; Sundararajan et al. 2012). Because of their potential in the detection of *lacZ* expression in embryos, we decided to test X-gal and Salmon-gal in combination with tetrazolium salts NBT, TNBT and INT as alternative substrates for β-galactosidase histochemistry on tissue sections of adult mouse brains. All combinations of substrates resulted in unambiguous readout of β-galactosidase activity in the regions of high expression in adult B6.Cg-Tg(Nes-cre)1Nogu mouse brains such as the thalamic and laterodorsal tegmental nuclei (Figs. 3, 5 and 7 are low-magnification images; Figs. 4 and 6 are high-magnification images of the corresponding nuclei). However, there were clear variations in the ability to reveal areas of lower β-galactosidase activity, the amount of background staining, and the color and speed of the staining reaction. The X-gal and NBT combination showed a clear dark blue stain almost devoid of background, and the reaction time was reasonably fast, up to 24 h (Figs. 3a, b, e, 4e, f; Table 1). This staining combination revealed the largest number of positive nuclei and cells in the B6.Cg-Tg(Nes-cre)1Nogu mouse brain, with the least background staining, visible only in the pyramidal cells of CA1–3 of the hippocampus, choroid plexus of the ventricles, and paraventricular nuclei of the hypothalamus (Fig. 3e). The X-gal/TNBT staining mixture produced clear dark brown precipitate in the same areas stained with the X-gal/NBT, but it also generated a higher level of background staining (Figs. 5a, b, e, 6a, b; Table 1). The background was kept to a minimum with shorter incubation time (up to 5 h), but achieving the same level of sensitivity as X-gal/NBT, a reaction time of up to 24 h was required. Under these conditions, background staining was considerably high, with labeled cells in all parts of the B6.Cg-Tg(Nes-cre)1Nogu and wild-type mouse brains (Fig. 5a, b, e). X-gal in combination with INT provided an orange-red staining pattern (Figs. 6e, f, 7a, b, e). This was the weakest combination of substrates, as it revealed some positive cells in the thalamic nuclei, but failed to clearly outline all that appeared positive with X-gal/NBT. Moreover, it needed the longest incubation time, up to 48 h, and resulted in the highest level of background. Background staining was characterized not only by precipitates in the cells, but also with large, needle-like brown crystals infiltrating whole sections of transgenic and wild-type brains (Fig. 7a, b, e). Among the tetrazolium salts used in this comparison review, INT was reported as yielding formazan deposits of the largest size and in the shape of needle crystals, and TNBT the smallest, diffuse granular deposits (Altman 1976).

The salmon-gal/NBT mix produced a dark purple precipitate with slightly higher background staining than X-gal/NBT, but the reaction time was much faster, up to 5 h (Figs. 3c, d, f, 4g, h). The sensitivity of the reaction was similar to that of X-gal/NBT (Table 1). Salmon-gal in combination with TNBT provided a dark brown precipitate, similar to X-gal/TNBT (Figs. 5c, d, 6c, d). While the reaction time (up to 5 h) was the same and the sensitivity of the reaction was similar to that obtained with Salmon-gal/NBT, the level of background staining was significantly higher (Fig. 5f). Similar to the X-gal/INT, the Salmon-gal/INT staining combination resulted in the highest background staining. The same diffuse deposits of needle-like brown crystals were seen in both the B6.Cg-Tg(Nes-cre)1Nogu and wild-type mouse brain sections (Figs. 6g, h, 7c, d, f). In some thalamic nuclei of the B6.Cg-Tg(Nes-cre)1Nogu mouse, positive cells marked with dark brick-red precipitate could be seen. The reaction time was considerably longer, up to 48 h, and similar to that with X-gal/INT.

## The mammalian β-galactosidase

It is well known that many mammalian cells and tissues exhibit endogenous β-galactosidase activity (Pearson et al. 1963; Lojda 1970; Shimohama et al. 1989; Renfranz et al. 1991; Hatton and Lin 1992; Weiss et al. 1997; Weiss et al. 1999; Sanchez-Ramos et al. 2000). Several isozymes of mammalian β-galactosidase have been described in various tissues, the most common of which is the lysosomal enzyme, important for the enzymatic degradation of glycolipids and mucopolysaccharides. Its pH optimum for activity is around 3.5–5.5. The second form of the endogenous β-galactosidase enzyme is primarily localized in the intestine and kidney, and has a pH optimum of 5.5–6.0 (Lojda 1970). The *Escherichia coli* β-galactosidase has neutral pH optimum of 7.3. X-gal-positive cells in the normal rat brain have been reported in the nuclei of the cranial nerves, red nucleus, inferior olivary nucleus, the magnocellular reticular formation, the region of the trapezoid body, medial habenula, paraventricular and supraoptic nuclei of the hypothalamus, substantia nigra, the Purkinje cell layer of the cerebellum, and other brain regions (Renfranz et al. 1991; Hatton and Lin 1992; Rosenberg et al. 1992; Sanchez-Ramos et al. 2000). When an appropriate buffer is used, with neutral or slightly alkaline conditions maintained throughout the whole staining, and an appropriate control is included, the detection of endogenous β-galactosidase activity may be significantly reduced (Rosenberg et al. 1992; Weiss et al. 1997; Weiss et al. 1999; Sanchez-Ramos et al. 2000). The use of ferric and ferrous ions in traditional staining solutions for the detection of *lacZ* gene expression in transgenic animals also leads to a reduction in activity of the endogenous acidic β-galactosidase (Lojda 1970). The substitution of tetrazolium salts may cause an increase in background staining because of the increased activity of the endogenous β-galactosidase. In addition, the reduction of tetrazolium salts to form insoluble colored formazan compounds can lead to the production of other chromogenic compounds, such as aniline (Altman 1976). The even higher background staining observed in the X-gal or Salmon-gal assay in combination with INT might be due to the acidification of the reaction mixture during the prolonged exposure of fixed tissues (incubation time for up to 48 h). In light of these risks, tetrazolium salts should be used with caution, and appropriate controls should be included for circumventing pitfalls and optimizing the reliability of this histochemical technique.

Overall, the most specific staining of B6.Cg-Tg(Nes-cre)1Nogu mouse brain sections was obtained with X-gal in combination with NBT (Table 1). Compared to Salmon-gal/NBT it produced darker staining, with much higher resolution and negligible background staining. The Salmon-gal/NBT staining mixture was the fastest, but with a slightly higher level of background staining. Based on the data collected (Table 1), we conclude that X-gal/NBT provides faster and more specific staining than the traditional X-gal combination with ferri- and ferro-cyanide. We recommend X-gal/NBT staining mixture as the first choice, but when faster results are needed while maintaining acceptable levels of noise, the Salmon-gal/NBT combination should be considered as an alternative.

In summary, when used correctly and with appropriate controls in place, the β-galactosidase histochemical reaction can be quite useful and cost-effective. The superior sensitivity and specificity of X-gal and Salmon-gal in combination with NBT further enhances its value, as it enables the detection of low levels of β-galactosidase activity. This could be extremely important in the characterization of *lacZ*-targeted knockout mice (Skarnes et al. 2011), which are readily available through the Knockout Mouse Project (KOMP) and the Knockout Mouse Phenotyping Project (KOMP2).
